# Geriatric day hospital: opportunity or threat? A qualitative exploratory study of the referral behaviour of Belgian general practitioners

**DOI:** 10.1186/1472-6963-10-202

**Published:** 2010-07-12

**Authors:** Piet Vanden Bussche, Fien Desmyter, Christiane Duchesnes, Valérie Massart, Didier Giet, Jean Petermans, Veerle Vyncke, Nele Ven Den Noortgate, Sara Willems

**Affiliations:** 1 Department of General Practice and Primary Health Care, Ghent University, UZ - 1K3, De Pintelaan 185, 9000 Gent, Belgium; 2 Department Geriatric Medicine, University Hospital Ghent, De Pintelaan 185, 9000 Gent, Belgium; 3 Centre Hospitalier Universitaire de Liège, Domaine Sart Tilman B35, 4000 Liège, Belgium; 4 Department of General Practice, University of Liège, avenue de l'Hôpital 3, 4000 Liège, Belgium

## Abstract

**Background:**

In order to address the challenges of an ageing population the Belgian government decided to allocate resources to the creation of geriatric day hospitals (GDHs). Although GDHs are meant to be a strategy to support general practitioners (GPs) caring for the frail elderly, few Belgian GPs seem to refer to a GDH. This study aims to explore the barriers and facilitating factors of GPs' referral to GDHs.

**Methods:**

A qualitative study using focus group discussions (FGDs) was conducted. Fifteen FGDs were organized in the different Belgian regions (Flanders, Wallonia, Brussels).

**Results:**

Contextual factors such as the unsatisfactory cooperation between hospital and GPs and organizational barriers such as the lack of communication on referral procedures between hospital and primary health care (PHC) were identified. Lack of basic knowledge about the concept or the local organization of GDH seemed to be a problem. Unclear task descriptions, responsibilities and activities of a GDH formed prominent points of discussion in all FGDs. Nevertheless a lot of possible advantages and disadvantages of GDHs for the patient and for the GP were mentioned.

**Conclusions:**

In the case of poor referral to GDHs, focusing on improving overall collaboration between primary and secondary health care is essential. This can be achieved by actively delivering adequate information, permanent communication and more involvement of PHC in the organization and functioning of GDHs. The absence of a transparent health care system with delineated role definitions, seems to hinder the integration of new initiatives like GDHs in the care process. Strategies to enhance referral to GDHs should use a comprehensive approach.

## Background

The organization and the quality of chronic care is an issue of growing importance in most countries [1,2]. Against the background of an ageing population with more complex care needs, the development of comprehensive, efficient, patient-centred, equitable and cost-effective health care is a true challenge [3].

International research shows that geriatric day hospitals (GDHs) are a potentially interesting tool in organizing comprehensive care for the elderly [4-8]. GDHs offer a specialized, multidisciplinary, individual and intensive ambulatory care for elderly patients within the setting of a hospital. This multidisciplinary approach may include assessment, treatment and rehabilitation. In a GDH several examinations and consultations are concentrated on one day in one location, avoiding long-term hospitalization [4,5,7-10]. The ultimate goal of the GDH is to contribute to the autonomy and the quality of life of the patient [8,11].

GDHs were first introduced in the '60 in the UK, as an important pillar of the care for the elderly. Since then, GDHs were also introduced in many other countries [12].

In Belgium a hospital centred 'Care Program for the Geriatric Patient' was gradually introduced in 2007 and financed by the federal government [13]. This program regulates and stimulates the further development of five aspects of geriatric hospital care: the geriatric ward, the geriatric consultation, the internal liaison function, the external liaison function and the GDH. There was no parallel initiative to financially support primary care for the geriatric patient.

Being the bridge between the hospital and the community, GDHs can play an important role in the realisation of seamless care of high quality for frail elderly. However, although interdisciplinary cooperation with primary health care is a key issue in the realisation of this ambition, questions can be asked about the extent to which GDHs are part of an integrated strategic multilevel (primary, secondary and tertiary care) approach of care for the elderly. International research shows that most GPs are unaware of GDHs, seldom visit a GDH or refer infrequently to it [14-16]. Recent evaluation studies in Belgium report similar findings: in a reference period of 3 months, only 40% (n = 800) of the patients in GDHs (n = 1.992) were referred by their GP [17]. After dismissal from the GDH, in 60% (n = 1.540) of the cases (n = 2.519) a follow-up was planned. 74% (n = 1.147) was referred back to the GDH or the policlinic consultation, only 19.5% (n = 301) were referred to the GP and 6% (n = 92) to somewhere else [9,10].

Little is known about the reasons why GPs are not referring to GDHs. In this study we want to explore the underlying factors and mechanisms determining why GDHs are so little used by Belgian GPs.

## Methods

### Design

Because of the exploratory character of the research question we selected a qualitative study design using focus group discussions (FGDs). A strength of FGD is the dynamic of the group; by discussing topics in group a process of sharing and comparing is installed [18].

### Sampling

Convenience sampling was used in order to reach maximal variance. To reach maximal geographical variance, research areas were selected based on geographical location, level of urbanization and the proximity of a GDH.

To maximise in depth analysis and discussion (a main objective of FGDs) the researchers tried to create a 'safe' environment. In the French speaking part of Belgium the number of GPs cooperating with a GDH was thought to be significantly higher than in the Dutch speaking part of Belgium. Also there is a clear difference in culture between the Northern and the Southern part of Belgium: in Flanders local quality peer review groups (QPRGs) are a regular channel for asking the opinion of GPs, used by the government, local policy makers and researchers. This is less the case in the French speaking areas. As a result, in the French area at hoc FDGs were organised for users and non-users separately. In Flanders the FGDs were organised in existing QPRGs without making a distinction between users and non-users.

To recruit GPs in each of the selected areas, the Dutch and French speaking research team adopted a slightly different approach. In the French speaking areas the official database of GPs was used to select participants. Because this database contains only the names and addresses of all GPs, further selection based on gender, age, years of experience,... was not possible. Therefore, a random selection of GPs was made. They were contacted by telephone and invited to participate in the study. To maximize participation and especially to guarantee participation of hard-to-reach GPs (i.e. GPs who usually do not respond to invitations for participation in studies), some participants were asked to invite and motivate their local colleagues.

In Flanders, in each of the five research areas the local QPRG was contacted. Once the chairman of the QPRG agreed to participate, the FGD was scheduled on the agenda of the next meeting of the QPRG.

A total of 15 FGDs were organized: 10 in the French speaking part of Belgium (the Walloon and Brussels region) and 5 in the Dutch speaking part (Flanders).

### Procedure

All FGDs were moderated by a native speaker (FD and CD), trained in FGDs. An interview guide including open-ended and semi-structured prompting questions covering the aspects of interest, was used. The topic guide was developed in cooperation between both research teams and evaluated by a multidisciplinary panel of methodological and content experts. All FGDs took between 1,5 and 2 hours. The number of participants per FGD varied from 3 to 10 GPs.

The FGD started with a short presentation of the research project (identification of the funder, background, aims, methods, timing). Next, all participants gave written informed consent and filled in a short questionnaire on demographics, practice characteristics and experience(s) with a GDH. In the course of some of the FGDs it became clear that GPs didn't know about the Belgian Care Program for the Geriatric Patient and/or the local situation. In that case, to enable participants to give a more balanced view on the topic, oral or written information on GDHs and their local situation (availability, number of GDHs, team composition) was provided. The discussions were audio taped and fully transcribed verbatim for coding and analyzing.

### Participant Characteristics

106 Belgian GPs participated. 83% were male and 17% were female (in Belgium dd. 31/12/2008 on a total of 13.049 GPs resp. 68.8% are male and 31.2% female) [personal communication with RIZIV Service for medical care]. There was variation in type of practice (solo, duo, group). A limited number of participants worked in a community health centre or as a GP in an emergency department of a hospital. The GPs' work experience varied between 2 and 48 years. Most GPs had no experience in referring to a GDH. Among the French speaking GPs (n = 63) those who declared having experience in referring to a GDH (n = 30) usually had a very limited experience (referred less than 5 times). The participant composition of the FGDs is shown in Table 1 and Table 2.

**Table 1:** Composition of the Dutch Speaking Focus Group Discussions (FGDs)

Region FGD	General Practitioner	Gender	Years of work experience	Setting	Estimated percentage of patients > 65 years
Oostkamp1	GP1	Male	23	Solo	30%
(n = 9)	GP2	Male	22	Solo	30%
	GP3	Male	28	Group	50%
	GP4	Female	3	Emergency room	20%
	GP5	Male	28	Group	30%
	GP6	Male	25	Solo	15%
	GP7	Male	32	Duo	33%
	GP8	Male	33	Duo	40%
	GP9	Male	6	Group	10%

Oostkamp2	GP1	Female	2	Duo	Missing
(n = 7)	GP2	Male	21	Solo	20%
	GP3	Male	17	Solo	15%
	GP4	Female	4	Emergency room	5-10%
	GP5	Female	2	Group	5%
	GP6	Male	7	Solo	30%
	GP7	Male	33	Solo	45%

Genk	GP1	Male	44	Duo	40%
(n = 10)	GP2	Male	22	Solo	Missing
	GP3	Male	29	Solo	Missing
	GP4	Male	25	Solo	25%
	GP5	Male	19	Solo	20%
	GP6	Male	23	Solo	40%
	GP7	Male	37	Solo	40%
	GP8	Male	33	Duo	50%
	GP9	Male	31	Solo	50%
	GP10	Male	28	Solo	30%

Ghent	GP1	Male	20	Duo	5%
(n = 8)	GP2	Male	48	Solo	75%
	GP3	Male	30	Duo	30%
	GP4	Male	21	Duo	15%
	GP5	Female	32	Solo	7%
	GP6	Male	22	Solo	40%
	GP7	Female	22	Duo	5%
	GP8	Female	15	Solo	50%

Deurne	GP1	Male	20	Community Health Centre	45%
(n = 9)	GP2	Male	34	Group	50%
	GP3	Male	26	Group	15%
	GP4	Male	15	Group	30%
	GP5	Male	28	Solo	20%
	GP6	Male	32	Duo	40%
	GP7	Male	26	Duo	20%
	GP8	Male	25	Duo	25%
	GP9	Male	24	Solo	30%

**Table 2:** Composition of the French Speaking Focus Group Discussions (FGDs)

Region FGD	Users or non-users of GDH	General Practitioner	Gender	Years of work experience	Setting	Estimated percentage of patients > 65 years	For users: number of referrals to GDH
Liège	Users	GP1	Male	25	Solo	40%	6-10
(n = 3)		GP2	Male	36	Solo	20 - 25%	> 10
		GP3	Male	28	Solo	25%	0-5

Liège	Non-users	GP1	Female	14	Solo	20%	n/a
(n = 6)		GP2	Male	20	Solo	20%	n/a
		GP3	Female	6	Group	25%	n/a
		GP4	Female	12	Group	30%	n/a
		GP5	Male	3	Group	25 - 30%	n/a
		GP6	Male	20	Solo	50%	n/a

Hainaut	Non-users	GP1	Male	Missing	Duo	Missing	n/a
(n = 7)		GP2	Male	28	Solo	60%	n/a
		GP3	Male	17	Solo	25%	n/a
		GP4	Male	28	Solo	50%	n/a
		GP5	Female	27	Duo	70%	n/a
		GP6	Male	27	Duo	15%	n/a
		GP7	Male	21	Solo	20%	n/a

Hainaut	Users	GP1	Male	30	Solo	30%	0-5
(n = 8)		GP2	Male	42	Duo	50%	0-5
		GP3	Male	28	Solo	20%	0-5
		GP4	Male	24	Solo	20%	0-5
		GP5	Male	33	Solo	40%	6-10
		GP6	Male	30	Solo	35%	6-10
		GP7	Female	25	Solo	25%	0-5
		GP8	Male	29	Solo	30%	0-5

Brussels	Users	GP1	Male	27	Solo	40%	0
(n = 4)		GP2	Male	47	Solo	35%	0-5
		GP3	Male	35	Solo	50%	0-5
		GP4	man	35	Solo	40%	> 10

Brussels	Non-users	GP1	Male	32	Solo	30%	n/a
(n = 3)		GP2	Male	39	Solo	> 50%	n/a
		GP3	Male	34	Solo	30%	0-5

Luxemburg	Users	GP1	Male	25	Solo	15%	0-5
(n = 6)		GP2	Male	30	Duo	25%	0-5
		GP3	Female	6	Duo	20%	0-5
		GP4	Male	24	Solo	40%	0-5
		GP5	Female	10	Solo	15 - 20%	0-5
		GP6	Male	11	Solo	30%	0-5

Luxemburg	Non-users	GP1	Male	35	Solo	60%	n/a
(n = 8)		GP2	Male	32	Solo	45%	n/a
		GP3	Male	19	Missing	25%	n/a
		GP4	Female	3,5	Missing	20%	n/a
		GP5	Female	14	Solo	30%	n/a
		GP6	Male	21	Solo	30%	n/a
		GP7	Male	32	Solo	30 - 40%	n/a
		GP8	Male	34	Solo	40%	n/a

Namur	Users	GP1	Male	15	Solo	35%	0
(n = 9)		GP2	Male	28	Solo	15 - 25%	6-10
		GP3	Male	35	Solo	35%	0-5
		GP4	Female	26	Solo	30%	0-5
		GP5	Female	30	Solo	30%	6-10
		GP6	Male	30	Solo	50%	6-10
		GP7	Male	31	Duo	50%	Missing
		GP8	Male	30	Solo	> 60%	Missing
		GP9	Male	34	Solo	40%	Missing

Namur	Non-users	GP1	Male	35	Solo	35%	n/a
(n = 9)		GP2	Male	38	Solo	30%	n/a
		GP3	Male	28	Solo	30%	n/a
		GP4	Male	25	Solo	30%	n/a
		GP5	Male	29	Solo	40%	n/a
		GP6	Male	30	Solo	30 - 40%	n/a
		GP7	Male	22	Solo	30%	n/a
		GP8	Male	40	Solo	80%	n/a
		GP9	Male	20	Solo	30 - 40%	n/a

### Coding and analysis

After two FGDs the two main researchers (FD and CD) first analysed the two transcripts independently. To do so, the principles of the framework approach, in which the pre-defined questions and objectives are used as boundaries for the coding, were applied [19]. The main goal of this study was to identify/understand the barriers and the enabling factors experienced by GPs to refer patients to a GDH. Within the boundaries formed by the aims of this study, open ended coding was applied to code the data. The aim of this coding was to make sure that all the FGD sections that are related under the same heading can be retrieved with ease afterwards. An associated aim is to make sure that the volume of data under each of these headings is both manageable and meaningful [20]. NVivo 7 and MS Word 2007 were used to do this.

Next, both researchers met several times to develop a consensus on the coding system. This classification system was then used to analyze the transcripts of the following FGDs.

After coding all transcripts, the coded data were analyzed by the two research teams independently each using the OSOP-method. This method involves reading through each section of data in turn and noting, on single sheet of paper (OSOP), all the different issues that are raised by the coded extracts, along with the relevant respondent IDs if possible (not for all coded extracts the respondent was identifiable) [20]. Each team consisted of minimum three researchers with different backgrounds (general practice, social sciences, psychology, methodology). The complete OSOP provided the research team with a summary of all the issues within the code and the IDs of the relevant respondents next to them (if available). The next step was axial coding in which the research team considered how the identified issues might group together in broader themes [20]. Consequently, both research teams met and presented the broader themes they identified. The analysis by both teams was very similar: they identified the same main themes and many of the more detailed aspects were similar in both parts of the country. The identified themes were discussed until consensus was reached about one set of main themes and about the underlying aspects of the themes. These main themes formed the backbone of the result section of this paper.

In the transcripts of the last two FGDs no new information occurred, suggesting that data saturation was reached.

### Ethics

This study was approved by the Ethics Committee of the University Hospital Ghent and the Comité d'Ethique Hospitalo-Facultaire Universitaire de Liège (EC registration number: B67020083854). Subsequently, the study was submitted at the Commission for the protection of privacy. This declaration was registered and published in the public register of the commission (PC processing number: VT005005950).

## Results

The analysis of the data showed a broad spectrum of barriers and possible facilitators to use the GDH as a tool in the care for the frail elderly.

The following major themes were identified: the lack of knowledge about the general concept of GDHs, the uncertainty about the necessity of this new initiative and its value in optimizing the care for the elderly, the lack of knowledge about local procedures and accessibility, the advantages and disadvantages for patients. Finally GPs also identified possible facilitators for cooperation and formulated suggestions on how to establish a sustainable relationship with GDHs.

Because of the participants' lack of knowledge and experience with GDHs, statements mentioned below are mainly based on GPs' assumptions and perceptions.

### Unknown, unloved

Most of the FGDs flagged at the very beginning because GPs had never heard of GDHs, did not know whether there was a GDH in their region or confused it with other services for geriatric patients. On top of this, there was a lack of specific easy-accessible information concerning admission, registration, functioning, legislation, financial aspects, planning and terminology of the GDH, both on local and national level.

*I had a completely different idea about it. I thought it concerned a day care centre for geriatric patients, where patients who live at home could get care during the day in a specially adapted environment.* (FGD Ghent, GP6)

*We do not know that it exists.* (FGD Arlon, Non users)

None of the GPs remembered the letter from the government that was sent to inform them about the start of GDHs. The few GPs who were familiar with the concept of GDHs learned about it unintended (e.g. a GP's patient was referred to a GDH by a geriatrician).

*This week I've just heard about geriatric day hospital for the first time. It was for an Alzheimer patient, someone who needed a CTscan. *(FGD Oostkamp1, GP2)

The very small number of GPs who already referred to a GDH, did this occasionally and consider it not as a natural reflex.

*One has to think of it.* (FGD Hainaut, Users)

### Doubts and questions about the position of the geriatric day hospital

In general the GPs emphasized the need for and the importance of good geriatric care, efficient assessment, sound diagnosis and shorter hospitalization. However they sometimes questioned the need for a new initiative like the GDH to reach these needs.

After the introduction of oral and/or written information about GDHs in the FGDs by the moderator, GPs recognized the relevance of the GDHs: they help to avoid or shorten hospitalization and might contribute to the return of the patient to his home setting by facilitating and prolonging his autonomy and quality of life. GDHs contribute to provide efficient assessment and diagnosis, however some GPs remained sceptical about the feasibility of a one-day-assessment. The evaluation function of a GDH could be valuable to a GP in case he/she needs additional input when making a diagnosis.

*I can imagine if a geriatric patient needs a triad of examinations, instead of travelling three times back and forth, it is possible in one day* (FGD Oostkamp1, GP2)

*I think it is positive because we often feel alone in our practice *(FGD Hainaut, Non users)

GPs were not convinced of the usefulness of therapeutic and rehabilitation programs of a GDH which require repeated contacts between patient and care provider.

*Rehabilitation on one day, that's not possible. It is a 'contradictio in terminis', I think.* (FGD Ghent, GP1)

Some GPs suggested that GDHs might prevent emergency admission to the hospital and decrease the pressure in the emergency rooms. Possible financial benefits for the social security system from the existence of GDHs by avoiding expensive and long-term hospitalizations were also mentioned. Some participants even agreed that they could contribute to avoiding or postponing residential care for the elderly.

*I think it is useful if one can organize many tests on the same day. Everybody could win from this. *(FGD Brussels, Users)

The GPs feel more confident with an accessible outpatients' clinic combined with short periods of hospitalization, and ad-hoc consultations of specialists in case they need backup or an second opinion.

*I have a network of specialists to whom I refer when I see a pathology. I have my preferences. I ask my patient: do you know someone to whom you wish to be referred to? No? Then I propose someone and I choose the specialist.* (FGD Ghent, GP6)

GPs also discussed the financial management of the GDH and the cost for the patient and society. According to some GPs the creation of GDHs is a strategy for secondary health care to benefit from extra funding.

*In fact, what interests me the most is: How much does it cost for the government? Why did they introduce this? For example, I can imagine that someone with a faecal incontinence can be referred to a GDH and then a coloscopy can be requested, but anyway, a faecal incontinence can be treated at a family practice with assistance of a nurse as well. So, I am wondering what it costs.* (FGD Ghent, GP5)

*I know that there is playing a very large commercial factor, that they try to take the national cake and that they try longer and harder to put us offside in all fields.* (FGD Oostkamp2, GP3)

*It is well known, the excess of examinations which we can request, it is to keep it going.* (FGD Brussels, Users)

### Lack of knowledge about local procedures and accessibility

A lot of questions arose concerning the modalities, entity, coordination and organization of a GDH. Knowledge of the basic facts as opening hours, contact person and admission routine was completely lacking. GPs wondered about the tasks and responsibilities of the different professional care givers within the hospital and their relation to primary care workers. There were remarks on the lack of clear role definition between geriatricians and other specialists in the hospital.

*In the existing GDH, who decides which tests are done? Is it the geriatrician or is it the doctor who referred? *(FG1)

*The problem is to know who to address. It is necessary to know to who I can refer, who is the coordinator at the hospital? *(FGD Hainaut, Non Users)

*I think that we can coordinate most of the requests for examinations and request adequately, but that the geriatrician has somehow the final responsibility, supervises and that he gives us feedback on the final report. *(FGD Oostkamp2, GP9)

*But I still do not understand the procedure. Do you say as a GP: "they have to get this examination and they have to get that..." Do I have to call the hospital and ask if they can fix everything and if the patient can be seen by the geriatrician? Does he decides what must happen and will the work be taken completely out of our hands or hmm... I still do not see what actually is happening there. Do people go there, are they taken care of by nurses, and are they transported to the necessary places where the examinations are carried out?* (FGD Ghent, GP8)

*I think it is important that we know which disciplines will be working in that clinic. *(FGD Genk, GP8) 

*What should they offer according to you?* (FGD Genk, M)

*Certainly a cardiologist, a gastroenterologist, an orthopaedic surgeon. *(FGD Genk, GP9)

A local effort of the GDHs should be undertaken to inform GP's about the overall concept and to discuss easy access and continuous information. A strongly profiled contact point, a dispatcher, a clear referral letter, correct follow-up and straight procedures were suggestions made by the GPs.

*We should be accurate when referring a patient. It is important to personally contact someone.... It is even better to go to the place.* (FGD Hainaut, Users)

*The first advantage could be that there is a very good referral letter, in which you request something specific. Such as: I really want a diagnosis, what exactly is the problem and that they stick to it. Now, the problem could be in the referral letter and there are a lot of examinations and they do not find the problem quickly. But I think that sometimes we want something very specific; that should be possible. And they should stick to it. And I think that we wish a fast response. That is what we expect.* (FGD Oostkamp2, GP5)

### For the benefit of the patient?

The participating GPs listed numerous advantages and disadvantages of a GDH for the patient. It is clear that avoiding hospitalization is a major advantage. A lot of physical and socio-psychological side effects such as hospital infections, alienation, disorientation and other complications resulting from being bedridden, can be avoided [21,22].

*It is important to keep in mind that hospitals are contaminated. It is always better to avoid being there. *(FGD Brussels, Non Users)

The elderly patient, often reluctant to enter the hospital, feels more at ease if he knows he can return home on the same day. In comparison with a longer stay in a hospital the patient's family must travel less. In a GDH the patient can be accompanied by a relative at all time and he is offered comfort and specialized individual care.

*I think it is also interesting for the family of the patient: they can continue the care, without being away from home or work.* (FGD Deurne, GP1)

A disadvantage is the number of examinations in one day. The GPs think they are not always necessary and may be too stressful and exhausting for elderly patients. However, good coordination of these examinations could help to reduce the strain on the patient. Respondents also indicated the potential transport problems of older patients who depend on their family or neighbours for getting to the hospital.

*It is perhaps even more tiresome for the patient if he must do the marathon between several doctors. That's not obvious. *(FGD Luxemburg, Users)

### "Help me to help my patient": supporting the general practitioner in his care for the geriatric patient

The GDH can support the GP in his care for the geriatric patient. In the FGDs potential advantages and necessary facilitators to lower the threshold for referral to the GDH were identified.

The GPs reported that by avoiding hospitalization the excess of examinations over which the GP has no control can be diminished.

Referring to a GDH gives the GP the possibility to generate a 'second opinion' from a specialist and a multidisciplinary team. However, this might be time-consuming since an appointment for admission has to be made, information needs to be transferred to the GDH and agreements on the dismissal have to be made.

*You can test your opinion and have it confirmed by someone else. If you work solo, you can jump very quickly to symptom treatment, certainly with the geriatric patient, and sometimes you can no longer see the pathology. In that case it is good that you can test this.* (FGD Deurne, GP7)

Some participants stressed the need for fast referral if they want to make use of a GDH. In some regions the long waiting time for admission is discouraging. The majority of the GPs regret the delay in reporting back which is threatening for the quality of the patient's follow-up.

*You think that the patient has dementia. Then you call for an appointment. Then he has to wait a couple of months, perhaps a bit longer. Then he can be seen once or a couple of times, then you have to wait again, one, two, or three months for the report. Well, after half a year approximately, then the patient can perhaps get medication.* (FGD Genk, GP2)

### Recognition of the role of the general practitioner is essential

A global dissatisfaction of the GP's position in the health care system was brought to the surface. Additionally, the GPs formulated suggestions on how to establish a sustainable relationship with GDHs.

By far the most discussed and prominent theme in all FGDs concerned the ambiguous relationship between GP and hospital in general and the GP's role in the care for the geriatric patient in particular. Primarily some participants experienced the development of GDHs as threatening. They feared secondary health care intruding into PHC. In this context, some GPs referred to the term 'hospitalocentrism'.

*All that fuss...you seldom see your patient ever again. You lose them, lifelong. I have my thoughts. It is a purely commercial matter. The hospital wants to have one's cake and eat it too and wants the victory as much as possible. Of course, there is certainly an indication and a place for it, but I think, and that's my biggest criticism on this matter and all that fuss, GPs are always the last ones to know. (*FGD Oostkamp2, GP8)

Participants felt that the GDH is a hospital structure that poorly responds to their needs. Lack of good cooperation and communication between hospitals and GPs is a frequently experienced problem. This is perceived as an ignorance of the GP's coordinating role and they fear that once the patient is referred, they lose contact with the patient.

*In the long run the GP will no longer see the patient. The GP refers to the geriatrician and the geriatrician coordinates. But then you no longer see your patient. (*FGD Ghent, GP5)

*We feel we lose patients and even our competences with this kind of institution.* (FGD Arlon, Non Users)

According to some GPs a geriatric day hospitalization and follow-up should be arranged on the GP's request and should not be in hands of the geriatrician (with the GP as a gatekeeper). Hospitals should take into account the GP's demands and patient's wishes. The GPs stressed their privileged personal relationship with the patient and their knowledge of the context and background of the patient which is insufficiently valued in hospital care. In case of planned diagnostics and assessment GPs asked for more goal-oriented cooperation and a patient-oriented approach.

*I expect from a geriatrician honesty that if you have a problem, he only focuses on that problem and not involves a whole set of tests. That's what I expect from him. It is a matter of professional ethics.* (FGD Oostkamp2, GP6)

*I think that there should be a large guarantee if you refer a patient. If you formulate a problem and you wish for a solution, you should be involved as much as possible. It should not be taken off hands and passed on to outpatients' clinics or all sorts of. So, if you formulate whichever question, you should get a clear answer by which you can manage, alone, without constant interaction with that service or other services. *(FGD Deurne, GP9)

*Always referral back, always seeing the patient again. Being involved in each step. That is a first guarantee we have, so that the patient does not disappear wherever to. And at each step we want to be informed. That should not be much, e.g. a phone message. That doesn't have to be a letter. As long as we know what has happened to the patient. Step by step. *(FGD Deurne, GP7)

Although the cultural background between Flemish and French speaking GP's was expected to differ, we seldom encountered differences on the various themes analysed. Only concerning this theme, Flemish GPs put more emphasis on participation and active involvement in developing the GDH services.

## Discussion

GPs have an increasing group of frail elderly among their patients. In theory, GDHs can support GPs in optimizing comprehensive care for them. However, national and international studies show that GPs and their patients seldom refer to a GDH [14-17]. Underlying factors preventing or facilitating the GP's referral behaviour to GDHs were identified by conducting 15 FGDs with 106 Belgian GPs. As far as we know, this is one of the first in depth analysis on the perception of GDHs by GPs.

Firstly, a lack of knowledge about GDHs was expressed prominently. GPs were not aware of the existence and the role of the GDHs. Consistent with the research of Williams et al. (1988) and according to Myint (2006), GPs confused the functions of the GDHs with hospital departments of geriatric medicine or day centres [16,23].

After giving them information on the concept and the local situation, the majority of the GPs were sceptical towards the concept of GDH, its aim and functioning. Questions were raised about the added value of the GDH as a new structure. In this context some of the participating GPs even identified a true mismatch between offered services and local needs of frail elderly and their GPs: the need for a quick, multidisciplinary, diagnostic assessment stands in contrast with the very long waiting times for admission to geriatric care in general, and the late reporting back after the patient is dismissed. Yet, Piterman and Koritsas (2005) showed that when the GP provides the specialist with information concerning the patient's medical history and when the specialist provides the GP afterwards with a clear report on the diagnosis, treatment and follow-up requirements, then both are more satisfied with the mutual referral process [24].

Secondly, most striking was the GPs irritation about not being involved in the development of the Care Program for the Geriatric Patient. A perceived lack of recognition for their key role was prominent in all FGDs. This discussion can partly be attributed to the lack of a gate keeping function by GPs in the Belgian health care system. However, also in countries with gate keeping positions for GPs, GPs are discontent with the (lack of) involvement in health care reforms and comprehensive care developments [3].

Literature shows that geriatric patients benefit from an evaluation, treatment or rehabilitation program in a GDH [25,26]. When GPs and their patients are convinced of the advantages of GDHs, referral habits might change. In this study the GPs recognised the advantage of a concentration of different services and health care providers in one facility. Not having to stay in the hospital overnight can lower the threshold for elderly who are often reluctant to enter the hospital. However, looking from the patient's perspective, GPs are afraid that the large number of examinations within a short time can be a burden for the older patient. GDHs should pay attention to these explicit demands. This corresponds with the findings of Khan (2009) suggesting that concentrating services in a day hospital, making different medical and paramedical staff available, providing fenced parking, transport, and toileting facilities under the same roof for frail elderly are the trump cards of the day hospital model [27].

### Implications for practice

Most GPs were sceptical to make use of GDHs and were unsure about the added value of this new system; a universal and well-known resistance to change was identified. At the same time the need for change in the organization of health care was prominently expressed in the FGDs.

The referral to GHDs is prevented because GPs feel threatened and forced to enter into an unfair "competition" with specialist care. A culture of mutual respect and trust between primary and secondary health care is a prerequisite for integrated care for the geriatric patient [28]. Government, policy makers, local hospitals and GPs have a major responsibility in this matter. On national level representatives of GPs should be asked to participate in the conception, the evaluation and the management of GDHs. The Chronic Care Model (CCM) describes how care could be organized differently, with the GP as the central hub and the secondary and tertiary care present to "support" PHC. By putting the GP in the middle of care, the CCM corresponds with the recommendations stated in the 2008 WHO Report "Primary Care Now More than Ever" [3].

To realise maximum implementation on the local level, hospitals and geriatrics have to inform local GPs and discuss with them the way the information can be shared. A repeated effort of the GDHs should be undertaken to inform GP's about the overall concept, to discuss easy access and to provide continuous information. A strongly profiled contact point, a dispatcher, a clear referral letter, correct follow-up and straight procedures were suggestions made by the GPs. Providing adequate information is an essential condition [29,30].

An important step onward is establishing a continuous communication strategy to enable the GDHs even to adapt to local and changing needs [27]. 

### Strengths and limitations of the study

The executed study is the first one exploring the perception of GDHs by GPs in a diverse and broad sample by means of a stringent qualitative method in both the Dutch speaking and French speaking region of Belgium. FGDs are effective to explore a plurality of information, but they have some limitations: the relative importance of each factor and explanation cannot be measured, the dynamic of the group can influence and limit the individual thoughts and the interviewer can have an effect on the group process. Another limitation of this study is the difference in recruitment strategies applied by both teams (mixed groups of GDH used in the Dutch groups vs. separate user and non-user groups in the French groups), prompted by cultural differences between the two regions. However, despite these differences, the outcome of the analysis in both groups were quasi parallel and unisonous.

Because of the strategy used to increase variation, it is not possible to determine a clear response rate. In Flanders 6 chairmen of QPRGs were contacted by telephone, 5 agreed to participate (one group had already scheduled another meeting). The attendance rate of the members to the QPRGs is about 80 percent. In the French speaking areas, to maximize participation of traditionally hard-to-reach GPs (i.e. GPs who do not respond to invitations for participation in studies), some at random selected participants were asked to invite and motivate their local colleagues. This makes it impossible to determine the response rate.

It is important to stress that both the secondary and tertiary care were not represented in the focus groups. Further research could focus on their experiences and how this influences referrals to GDHs.

We identified the urgent need for the implementation of an evidenced-based comprehensive and integrated health policy to tackle upcoming problems of the aging population and more specifically the frail elderly. Further evaluation of the effect of actions described in the 'implications for practice' paragraph, would be interesting and can support this process.

Additional in-depth study of the needs and expectations of primary and secondary care givers and the patients' opinions is essential and promising.

## Conclusions

Our findings emphasize the importance of a coherent health care system with a central role for the GP and both shared coordination, good collaboration and communication between primary and secondary health care. If not, this causes frustration, tension and lack of faith in new initiatives like GDHs.

Introducing new approaches and changing behaviour can only be achieved using an interdisciplinary bottom-up approach. All stakeholders (including patients) should be given the opportunity to participate in new developments. By creating the essential conditions of informing, stimulating and involving GPs in the matter of GDHs, both on national and local level, a shift towards successful use of patient-centered and multidisciplinary care units can be achieved.

## Competing interests

NV is the head of the GDH at the University Hospital Ghent.

JP is the head of the GDH at the Centre Hospitalier Universitaire de Liège.

PV and DG are GPs. The other authors declare not to have non-financial competing interests.

All authors declare not to have financial interests.

## Authors' contributions

NV, JP and SW coordinated the overall study, concept and design. VM, CD, FD and VV were responsible for the gathering of the subjects and data, the analysis and interpretation of the data and the preparation of the final report. SW, PV and DG contributed to the recruitment of the subjects, the analysis and interpretation of the data and the preparation of the final report. FD, SW and PV wrote this article.

FD and PV contributed equally to this work.

## Pre-publication history

The pre-publication history for this paper can be accessed here:

http://www.biomedcentral.com/1472-6963/10/202/prepub
